# Effects of starter nitrogen fertilizer on soybean root activity, leaf photosynthesis and grain yield

**DOI:** 10.1371/journal.pone.0174841

**Published:** 2017-04-07

**Authors:** Zhijia Gai, Jingtao Zhang, Caifeng Li

**Affiliations:** 1 Department of Agronomy, Northeast Agricultural University, Harbin, Heilongjiang, China; 2 Jiamusi Branch, Heilongjiang Academy of Agricultural Sciences, Jiamusi, Heilongjiang, China; Tennessee State University, UNITED STATES

## Abstract

The objective of this study was to examine the impact of starter nitrogen fertilizer on soybean root activity, leaf photosynthesis, grain yield and their relationship. To achieve this objective, field experiments were conducted in 2013 and 2014, using a randomized complete block design, with three replications. Nitrogen was applied at planting at rates of 0, 25, 50, and 75 kg N ha^-1^. In both years, starter nitrogen fertilizer benefited root activity, leaf photosynthesis, and consequently its yield. Statistically significant correlation was found among root activity, leaf photosynthetic rate, and grain yield at the developmental stage. The application of N_25_, N_50_, and N_75_ increased grain yield by 1.28%, 2.47%, and 1.58% in 2013 and by 0.62%, 2.77%, and 2.06% in 2014 compared to the N_0_ treatment. Maximum grain yield of 3238.91 kg ha^-1^ in 2013 and 3086.87 kg ha^-1^ in 2014 were recorded for N_50_ treatment. Grain yield was greater for 2013 than 2014, possibly due to more favorable environmental conditions. This research indicated that applying nitrogen as starter is necessary to increase soybean yield in Sangjiang River Plain in China.

## Introduction

There is speculation that supplied nitrogen from symbiotic N_2_-fixation is not always adequate to maximize soybean grain yield [1]. In the northeast of China, cool soil temperature at time of planting can limit soil microbial activity, and therefore potentially delay nitrogen fixation and possibly the vegetative growth in early stage. There are many factors influencing soybean nitrogen fixation and the response to applied nitrogen fertilizer. Sorensen et al. [2] reported that soil pH, temperature, and moisture affect soybean response to applied nitrogen fertilizer.

Nitrogen is one of the major nutrients that are required for soybean growth and development. Soybean plants obtain nitrogen from three sources [3], 1) nitrogen derived from biological N_2_-fixation by root nodule, 2) nitrogen requirement of soybean can be met by soil nitrogen [4]. High levels of soil nitrogen inhibit symbiotic N_2_-fixation, and under these conditions the soil supplies the majority of the plant's nitrogen needs [4]. Conversely, N_2_-fixation supplies the majority of the plant's nitrogen requirements under conditions of low soil nitrogen, and 3) nitrogen from applied fertilizer. For optimum soybean yield, it is necessary to use both biological N_2_-fixation and nitrogen uptake by soybean roots [5, 6]. Nitrogen fertilizer applied to soybean is based on the plant nitrogen needs during seedling development prior to nodule formation that is crucial to the growth and development of soybean [5, 7]. Hardy et al. [8] reported that N_2_-fixation began 14 days after planting only when soybean was cultivated under optimum temperature and moisture conditions, thus a small amount of nitrogen fertilizer at planting might be beneficial to early vegetative growth. In the study by Bergersen [9], it was pointed out that nitrogen applied before sowing was beneficial to soybean growth, given that soybean root nodules were not formed until at least 9 days after soybean emergence. Additionally, starter nitrogen fertilizer can supply nitrogen until biological N_2_-fixation begins by the root nodule [10].

Many researchers have extensively examined the effects of nitrogen fertilizer on soybean grain yield. Application of starter-N is directed at providing soybean with readily available soil nitrogen during seedling development, and has been shown to increase soybean grain yield [10]. However, nitrogen fertilizer at the time of planting may reduce N_2_-fixation of soybean and its yield [11]. Results from previous researchers have been contradictory. Sij et al. [12], in Texas reported that starter nitrogen had no effect on soybean plant height, shoot fresh weight, leaf area, or grain yield. However, the research conducted by Starling et al. [13] for soybean following corn in southern Alabama indicated that grain yield and plant growth were higher when nitrogen fertilizer was applied as starter. Furthermore, their study suggested that early application of starter nitrogen fertilizer had significant effects on soybean grain yield. In addition to soybean grain yield, starter fertilizer-N increased plant height and biomass in the study by Starling et al. [13]. Jerrery et al. [14] pointed out that a large amount of fertilizer-N significantly increased soybean grain yield for irrigated and non-irrigated environments, and N_2_-fixation may limit grain yield of soybean grown in both irrigated and non-irrigated environments of the midsouthern USA. Field experiments conducted by Boroomandan et al. [15] indicated that density of 45 plant m^-2^ and low level of nitrogen starter fertilizer (40 kg N ha^-1^) were optimum and increased soybean grain yield under conditions of their experiment. Research conducted in cool environments of the northern Great Plains indicated that low rates (<15 kg N ha^-1^) of starter fertilizer-N at planting increased soybean grain yield compared to no application of nitrogen fertilizer in 9 out of 11 years [16].

Researchers have determined the effects of nitrogen fertilizer on soybean grain yield, but results have been inconclusive. The effects of nitrogen fertilizer on soybean root nodules have been reported extensively. However, little information is available on the impacts of starter nitrogen fertilizer on leaf photosynthesis, root activity, and their relationship with grain yield. More specific information is needed on how nitrogen fertilizer influences soybean grain yield. Photosynthesis is a main pathway for plants to produce dry matter and it is estimated that 75 to 95% of crop dry weight is derived from photosynthesis [17]. Nitrogen derived from fertilizer in this study probably affect soybean root activity, leaf photosynthesis and consequently its yield. The objective of this investigation was to examine the relationship among root activity, photosynthetic parameters and soybean grain yield (not inoculated), with an emphasis on responses of root activity, leaf photosynthesis to starter nitrogen fertilizer rate in the 2-yr experiment in cool environments of Sangjiang River Plain of China.

## Materials and methods

A 2-year field study was conducted in 2013 and 2014 at Agricultural Experiment Station of Jiamusi Branch of Heilongjiang Academy of Agricultural Sciences, China (46°18’N, 130°22’E). The research site is located in the north temperate zone and continental monsoon area (rainy and hot in summer, cold and arid in winter), has an average annual precipitation of 510 mm, and an average available accumulated temperature (≥10°C) is 2521°C. The site soil is the typical Mollisol (black soil) with silty clay loam texture. Table 1 lists the monthly weather data for the 2013 and 2014 growing seasons. Soil samples were collected to a depth of 20 cm at each site prior to planting. Table 2 lists the characteristics of the soil at the experimental site in 2013 and 2014.

**Table 1:** Monthly weather data for the 2013 and 2014 growing seasons.

Month	2013		2014	
	Total precipitation (mm)	Mean air temperature (°C)	Total precipitation (mm)	Mean air temperature (°C)
May	55.1	15.2	129.0	13.5
June	49.6	21.0	72.0	21.4
July	220.4	22.5	72.4	22.4
August	127.7	21.2	53.8	21.1
September	42.4	14.5	89.9	14.7

**Table 2:** Characteristics of the topsoil (0–20 cm) before the experiment.

	pH	Organic matter (%)	Available N (mg kg^-1^)	Available P (mg kg^-1^)	Available K (mg kg^-1^)
2013	6.85	2.58	128.87	36.62	126.71
2014	6.71	2.31	109.63	38.46	115.69

The experiment was conducted as a randomized complete block (RCB) design with three replications. Urea (46-0-0) was the N source applied at rates of 0, 25, 50, and 75 kg N ha^-1^ identified as N_0_, N_25_, N_50_, and N_75_, respectively. Phosphorus and potassium were applied at planting to each plot at 40 kg P ha^-1^ as triple super-phosphate (0-36-0) and 20 kg K ha^-1^ as KCl (0-0-60). All fertilizers were applied at planting in band (5 cm below and 5 cm to side of the seed row). Plots were 3.6 ×10 m with a 0.45-m row spacing (8 rows). The previous crop was corn.

Soybean cultivar Henong42 was planted at a rate of 450000 seeds ha^-1^ by planter. Soybean was not inoculated before planting. Pendimethalin at 1.2 kg a.i. ha^-1^ plus paraquat at 1.2 kg a.i. ha^-1^ were applied to the entire experimental plots immediately after planting. Paraquat was applied to kill existing weeds at sowing. Pendimethalin was used to control early-season weeds. Haloxyfop-P at 0.05 kg a.i. ha^-1^ plus fomesafen at 0.25 kg a.i. ha^-1^ were applied at 4–5 weeks after planting to all the experimental plots for postemergence weed control. Haloxyfop-P was applied to kill gramineae weeds, and fomesafen was used to control broadleaf weed. Herbicides were applied with a tractor-mounted sprayer. Each plot was weeded by hand periodically throughout the season to keep weed-free. Soil sampling, crop planting, herbicide application, tillage, plant sampling, and harvest dates are reported in Table 3.

**Table 3:** Planting, herbicide application, plant sampling, harvest, and soil sampling dates, 2013–2014.

Agronomic practice	2013	2014
Fall chisel *	4 November	12 November
soil sampling	28 April	30 April
seedbed preparation	1 May	4 May
planting	5 May	7 May
V4 biomass	26June	29 June
R2 biomass	16July	18July
R4 biomass	6 August	10 August
Pre-emergence herbicide application	11 May	13 May
Post-emergence herbicide application	14 June	18 June
Harvest	21 September	19 September

*performed the previous fall

Samples were collected three times at V4 (completely unrolled leaf at the fourth node on the main stem), R2 (full bloom stage), and R4 (full pod stage) stages. Five consecutive plants were selected to determine the leaf area per plant, leaf dry weight per plant, leaf N yield, leaf area index (LAI), photosynthetic rate (Pn), and chlorophyll content (SPAD) of the middle leaflet of the third trifoliate leaf.

Leaf area per plant was determined by using Portable Leaf Area Meter AM-300 (Bio-Scientific, Ltd., Great Am well, Herefordshire, England) and following equation was used to calculate leaf area index: LAI = Leaf area per plant (cm^-2^)/Plant ground area (cm^-2^). The fresh leaves per plant were oven-dried at 60°C, to a constant weight, and then dry leaf weight was determined. Specific leaf weight (SLW) was calculated as the ratio of leaf dry weight per plant to leaf area per plant. Leaf samples were ground to pass through a 1-mm sieve. Total N yield in leaf was estimated by the Kjedahl method of Liu et al. [18]. Photosynthetic rate of the middle leaflet of the third trifoliate leaf was determined by using the CI-340 portable photosynthesis measuring system (CID, Inc., USA). Photosynthetic measurements were made on fully expanded leaves. Chlorophyll content (SPAD) of the middle leaflet of the third trifoliate leaf was assessed using SPAD-502 Chlorophyll Meter Model (Konica Minolta Inc., Japan).

Root activity was determined by using the triphenyl tetrazolium chloride (TTC) method [19]. The triphenyl tetrazolium chloride is a chemical that is reduced by dehydrogenases mainly succinate dehydrogenase, when added to a tissue. The dehydrogenase activity is regarded as an index of soybean root activity. Root activity = amount of TTC reduction (μg)/fresh root weight (g) × time (h). The roots per plant were carefully rinsed with tap water, and then oven-dried at 60°C, to a constant weight, and then the dry root weight was determined.

The yield components were pod number per plant, grain number per plant, grain number per pod, 100-grain weigh. Grain yield was determined by harvesting the plants in the two central rows from each plot. Harvested grains were weighed and adjusted to 130 g kg^-1^ moisture.

All the data were statistically analyzed using Proc Mixed in SAS. Nitrogen effects were considered fixed and years were treated as random effects. Mean comparisons were made under Fisher’s protected LSD Test at the 0.05 level of probability. Correlation analyses were also performed to evaluate the degree and significance of the correlation.

## Results and discussion

### Response of yield and yield components to starter nitrogen fertilizer

Grain yield and yield components under different nitrogen treatments were summarized in Table 4. Grain yield showed significant responses (P<0.05) to year and starter nitrogen fertilizer in 2014. The difference among N rates was significant, and N_50_ produced the highest grain yield in 2014 (Table 4). Grain yield at N_50_ was significantly higher than N_0_, and N_25_ treatments, but yield difference between N_50_ and N_75_ was not significant in 2014. Yield increase with applying starter fertilizer-N to soybean in 2014 was due to lower available N in the soil (Table 2). However, significant response of grain yield to starter nitrogen fertilizer in 2013 was not detected (Table 4). Individual treatment effects showed that the application of N_25_, N_50_, and N_75_ increased grain yield by 1.28%, 2.47%, and 1.58% in 2013 and by 0.62%, 2.77%, and 2.06% in 2014 over the control. Maximum grain yields of 3238.91 kg ha^-1^ for 2014 growing season and 3086.87 kg ha^-1^ in 2013 were recorded for N_50_ treatment. However, significant yield difference between N_50_ and N_75_ was not found in both years. There was significant difference between years for grain yield (P<0.05). The lower grain yield for 2014 growing season, compared with 2013 could be attributed to differences in precipitation throughout the growing season (Table 1). It has been shown that adequate rainfall was critical to optimal soybean grain yield [20, 21]. Serraj et al. [22] found that dry weather during the soybean growth season caused negative effects on soybean grain yield.

**Table 4:** Effects of starter nitrogen fertilizer on soybean yield and yield components on two field locations in 2013 and 2014.

Nitrogen treatments	Grain yield (kg ha^-1^)			Pod number per plant			Grain number per pod			Grain number per plant			100-grain weight (g)		
	2013	2014	Mean	2013	2014	Mean	2013	2014	Mean	2013	2014	Mean	2013	2014	Mean
N_0_	3160.98	3003.60c	3082.29	21.33	19.27b	20.30	2.67	2.66	2.67	56.87	51.13c	54.00	17.92	17.86	17.89
N_25_	3201.37	3022.31bc	3111.84	21.33	20.13b	20.73	2.68	2.58	2.63	57.13	51.87bc	54.50	18.00	17.96	17.98
N_50_	3238.91	3086.87a	3162.89	21.53	21.60a	21.57	2.70	2.50	2.60	58.00	53.67a	55.84	17.98	17.85	17.92
N_75_	3210.85	3065.56ab	3138.21	21.20	19.80b	20.50	2.72	2.66	2.69	57.33	52.73ab	55.03	17.94	17.87	17.91
ANOVA Effect															
Nitrogen (N)	ns	*	ns	ns	**	ns	ns	ns	ns	ns	*	ns	ns	ns	ns
Year (Y)	***			**			ns			***			ns		
N×Y	ns			ns			ns			ns			ns		

Means with same letter are not significantly different (P<0.05) using LSD Test.

***, **, *, and ns denote significance at 0.001, 0.01, 0.05 and not significant (P>0.05), respectively.

Soybean grain yield is directly influenced by many environmental factors. Among these, nitrogen fertilization can affect soybean grain yield [23]. Nitrogen is one of the most important nutrients affecting soybean grain yield [24–26]. Muchow et al. [27] reported that nitrogen input was a “major constraint” to high soybean yield based on analysis of crop stimulation. Maximum grain yield was obtained at intermediate level of nitrogen application each year in our study. Results from this study proved that excessive or insufficient nitrogen fertilizer was not beneficial to an increase in grain yield of soybean, and intermediate level of starter nitrogen fertilization (N_50_) increased grain yield. Similarly, an increase in grain yield of soybean has been observed in response to N application [28]. Our results are in agreement with the work of Tian [28], who pointed out that soybean grain yield at 1.06 g N pot^-1^ was significantly higher than that at 0 g N pot^-1^ and 1.59 g N pot^-1^ in the pot experiment. Based on their research, high level of nitrogen application reduced grain yield due to impeding the growth of soybean root and root nodule. However, Maw et al. [29] reported that four rates of N fertilizer (0, 25, 50, and 75 kg N ha^-1^) were applied as starter nitrogen and the highest grain yield in both seasons was observed at N_75_. A study conducted in Texas showed that nitrogen fertilizer applied at the time of planting had no significant effect on soybean leaf area or grain yield [12]. Some researchers believe that a small amount of starter fertilizer-N placed near the seed can cause a positive effect on early growth and soybean grain yield, particularly in situations where soil fertility is low or unfavorable environmental conditions exist [12]. Unfavorable conditions, such as cool climates, can lead to excessively cool soil temperature thus delaying biological N_2_-fixation [8].

There were significant differences between two years for pod number per plant and grain number per plant (P<0.05). Similar to the effect of starter nitrogen on grain yield in 2014, pod number per plant and grain number per plant showed significant response to starter nitrogen fertilizer for 2014 growing season (Table 4). Individual treatment effects illustrated that the application of N_25_, N_50_, and N_75_ increased pod number per plant by 4.46%, 12.09%, and 2.75% over the control in 2014, respectively. Our results showed that grain per plant at N_25_, N_50_, and N_75_ was 1.45%, 4.97%, and 3.13% higher compared to N_0_ in 2014. However, grain number per pod and 100-grain weight were not significantly affected by N fertilizer application each year and across years (P>0.05). Significant nitrogen by year interaction was not observed for yield components. Our results are in agreement with Sorensen et al. [2], Jeffery et al. [14], and Purell et al. [30], who found that the majority of soybean grain yield increase resulting from high rates of applied nitrogen fertilizer to soybean before planting is attributable to an increase in grain number. Pod number per plant was the yield component easily affected by change in environmental and cultural conditions [29, 31, 32]. In our experiment, starter nitrogen fertilizer had significant impacts on pod number per plant in 2014, while starter nitrogen exerted no significant effect on grain number per pod in 2014. Therefore, the increase in grain number per plant from starter nitrogen could be attributable to pod number per plant rather than grain number per pod (Table 4). Herbert [33] reported that grain number per pod was a minor component determining soybean grain yield. The small change of grain number per pod and 100-grain weight under different treatments indicated that grain number per pod and 100-grain weight were strongly determined by the internal genetic mechanism, and were less influenced by starter nitrogen fertilizer in our research. Similar to our results, Di et al. [34] pointed out that nitrogen had no significant influence on 100-grain weight. Jeffery et al. [14] reported that fertilizer N applied shortly after planting had little effect on grain weight in either the non-irrigated or irrigated environment. However, positive effect of nitrogen fertilizer on 100-grain weight was reported by several researchers [35–38].

### Response of dry root weight and root activity to starter nitrogen fertilizer

As shown in Tables 5 and 6, treatments significantly influenced dry root weight and root activity (P<0.05) at V4, and R2 stages each year and across years. Intermediate level of nitrogen fertilizer (N_50_) produced highest dry root weight and root activity, and thus highest grain yield in our study. Our results were in agreement with the work of Liu et al. [18], who found that high level of nitrogen fertilizer inhibited the growth and development of soybean root in the pot experiment. Wang et al. [39] also pointed out that a certain amount of nitrogen fertilizer benefited the increase of root weight. Our findings supported Zhang et al. [40], who reported that high nitrogen level inhibited the dry root weight and root activity at V5, R1, and R3 stages. Seasonal air temperature and precipitation patterns differed between 2013 and 2014 (Table 1). Therefore, there was significant difference between years for dry root weight and root activity.

**Table 5:** Effects of nitrogen treatments on dry root weight at different stages (g plant^-1^).

Nitrogen treatments	V4			R2			R4		
	2013	2014	Mean	2013	2014	Mean	2013	2014	Mean
N_0_	0.26c	0.29c	0.28d	1.38d	1.63c	1.51d	3.01	3.05c	3.03c
N_25_	0.36a	0.36b	0.36b	1.87b	1.88b	1.88b	3.11	3.25b	3.18b
N_50_	0.38a	0.45a	0.42a	1.96a	2.08a	2.02a	3.18	3.37a	3.28a
N_75_	0.31b	0.33b	0.32c	1.51c	1.83b	1.67c	3.07	3.17b	3.12b
ANOVA Effect									
Nitrogen (N)	**	**	***	*	**	**	ns	*	**
Year (Y)	**			**			**		
N×Y	*			*			ns		

Means with same letter are not significantly different (P<0.05) using LSD Test.

***, **, *, and ns denote significance at 0.001, 0.01, 0.05 and not significant (P>0.05), respectively.

**Table 6:** Effects of nitrogen treatments on root activity at different stages (TTC reduction μg g^-1^ h^-1^).

Nitrogen treatments	V4			R2			R4		
	2013	2014	Mean	2013	2014	Mean	2013	2014	Mean
N_0_	78.3c	72.5d	75.4d	129.8c	123.3c	126.6d	105.4	99.2c	102.3b
N_25_	80.3c	79.6c	80.0c	131.7bc	127.8c	129.8c	110.0	102.3bc	106.2b
N_50_	96.6a	94.1a	95.4a	148.4a	143.1a	145.8a	125.5	113.2a	119.4a
N_75_	83.8b	84.2b	84.0b	134.5b	133.2b	133.9b	111.4	108.4ab	109.9b
ANOVA Effect									
Nitrogen (N)	**	***	***	**	***	**	ns	*	**
Year (Y)	**			**			*		
N×Y	*			ns			ns		

Means with same letter are not significantly different (P<0.05) using LSD Test.

***, **, *, and ns denote significance at 0.001, 0.01, 0.05 and not significant (P>0.05), respectively.

### Response of leaf nitrogen yield to starter nitrogen fertilizer

As shown in Table 7, the leaf nitrogen yield varied significantly among treatments at V4, and R2 stages each year and across years. However, significant response of leaf N yield to starter nitrogen fertilizer was not seen at R4 stage in both years. The nitrogen yield in leaf increased with increasing nitrogen rates up to N_50_, and then decreased with applying higher rates of nitrogen fertilizer at the developmental stage. Nitrogen fertilizer at planting was beneficial to the increase in leaf nitrogen yield in our study. In terms of nutrient acquisition, the roots have long been recognized as being important in determining the capacity for plants to access and mediate the availability of essential nutrients in soil [41]. Our results were similar to the work of Osborne et al. [42] reported that starter nitrogen fertilizer had a positive impact on early plant nitrogen concentration at the early growth stage. High level of nitrogen fertilizer inhibited the root growth and root activity (Tables 5 and 6), and thus decreased the uptake and assimilation of nitrogen at the developmental stage in our study. Our results are in agreement with the previous experiment conducted by Wang et al. [39] pointed out that a certain amount of nitrogen fertilizer was beneficial to the soybean root growth and thus increased nitrogen uptake, especially in the soil with low available nitrogen.

**Table 7:** Effects of starter nitrogen fertilizer on N yield in leaf at different stages (g m^-2^).

Nitrogen treatments	V4			R2			R4		
	2013	2014	Mean	2013	2014	Mean	2013	2014	Mean
N_0_	0.693c	0.701b	0.697c	1.613c	1.570d	1.592d	3.346	3.262	3.304
N_25_	0.831b	0.758b	0.795b	1.697c	1.774c	1.736c	3.377	3.322	3.35
N_50_	0.930a	0.947a	0.939a	2.239a	2.337a	2.288a	3.366	3.212	3.289
N_75_	0.955a	0.899a	0.927a	1.931b	1.988b	1.960b	3.397	3.332	3.365
ANOVA Effect									
Nitrogen (N)	***	**	***	***	***	***	ns	ns	ns
Year (Y)	ns			ns			ns		
N×Y	ns			ns			ns		

Means with same letter are not significantly different (P<0.05) using LSD Test.

***, **, and ns denote significance at 0.001, 0.01and not significant (P>0.05), respectively.

### Response of chlorophyll content to starter nitrogen fertilizer

The data in Table 8 show that the chlorophyll content, in general, was positively affected by starter nitrogen fertilizer in 2013 and 2014. Starter nitrogen fertilizer significantly increased the chlorophyll content (SPAD value) at early stage (V4, and R2). The difference between years was significant. Similar to the early findings of Shen et al. [43], who reported that nitrogen applied at planting was beneficial to the increase in the chlorophyll content. High level of nitrogen fertilizer (N_75_) significantly decreased the chlorophyll content compared to intermediate level of nitrogen fertilizer (N_50_) at V4, and R2 stages in 2013. However, the difference in the chlorophyll content between N_75_ and N_50_ was not significant in 2014. That was probably because the available N of soil in 2013 was higher compared with 2014. High nitrogen level (N_75_) provided excessive nitrogen to the soil in 2014 (Table 2), inhibiting the root activity and dry root weight (Tables 5 and 6). Therefore, our results indicated that a heavy supply of fertilizer N applied to the soil with high available nitrogen could cause a decrease in the chlorophyll content.

**Table 8:** Effects of starter nitrogen fertilizer on leaf chlorophyll content at different stages.

Nitrogen treatments	V4			R2			R4		
	2013	2014	Mean	2013	2014	Mean	2013	2014	Mean
N_0_	40.52d	39.63b	40.08d	45.76b	44.36b	45.06b	40.62	39.90	40.26
N_25_	45.73b	41.21a	43.47b	48.98a	46.89b	47.94a	40.86	40.20	40.53
N_50_	47.19a	41.84a	44.52a	48.25a	49.98a	49.12a	40.72	40.71	40.72
N_75_	42.60c	41.39a	42.00c	47.89b	50.14a	49.02a	40.55	40.64	40.60
ANOVA Effect									
Nitrogen (N)	***	*	***	**	**	***	ns	ns	ns
Year (Y)	***			*			***		
N×Y	***			**			ns		

Means with same letter are not significantly different (P<0.05) using LSD Test.

***, **, *, and ns denote significance at 0.001, 0.01, 0.05 and not significant (P>0.05), respectively.

### Response of leaf photosynthetic rate to starter nitrogen fertilizer

The data in Table 9 indicated that there was significant difference between years for photosynthetic rate (P<0.01). Significant effect of nitrogen fertilizer on photosynthetic rate was detected at V4, and R2 stages each year, and starter nitrogen fertilizer had significant effect on photosynthetic rate at R4 stage in 2014. However, significant response of photosynthetic rate to fertilizer N was not found at R4 stage in 2013. That was largely due to the growing season environment at R4 stage. The photosynthetic rate is an important parameter of photosynthesis and enhancing photosynthetic rate is an important way to increase soybean grain yield [44]. Chlorophyll is vital for photosynthesis, which allows soybean to absorb energy from light [25, 45]. Our results showed that starter nitrogen fertilizer had positive effect on the chlorophyll content, and thus promoted the increase of photosynthetic rate at the developmental stage. Results from Zhang et al. [46] indicated that fertilizer N increased the chlorophyll content and photosynthetic rate of soybean in the pot experiment. Anten [47] reported that the improvement of photosynthetic characteristics has a significant impact on crop yield, growth and development. In our study, N_50_ produced highest grain yield, which could be ascribed to highest leaf photosynthetic rate at the developmental stages.

**Table 9:** Effects of starter nitrogen fertilizer on leaf photosynthetic rate at different stages (μmol CO_2_ m^-2^ s^-1^).

Nitrogen treatments	V4			R2			R4		
	2013	2014	Mean	2013	2014	Mean	2013	2014	Mean
N_0_	18.79bc	15.78b	15.90b	24.87a	22.20c	23.54b	20.23	17.35c	18.79
N_25_	19.91ab	16.84b	17.98b	25.06a	22.11c	23.59b	20.54	17.53bc	19.04
N_50_	21.13a	19.07a	19.29a	25.91a	26.24a	26.08a	20.51	18.11a	19.31
N_75_	17.93c	18.14a	16.35b	22.54b	24.27b	23.41b	20.06	17.84ab	18.95
ANOVA Effect									
Nitrogen (N)	*	*	***	**	**	***	ns	*	ns
Year (Y)	***			**			***		
N×Y	*			***			ns		

Means with same letter are not significantly different (P<0.05) using LSD Test.

***, **, *, and ns denote significance at 0.001, 0.01, 0.05 and not significant (P>0.05), respectively.

### Response of specific leaf weight to starter nitrogen fertilizer

The effect of N fertilization on specific leaf weight was shown in Table 10. Specific leaf weight, the ratio of leaf dry matter to leaf area, is an important parameter of evaluating photosynthetic capacity and is regarded as an important indicator for soybean variety selection by breeding researchers [48]. There was significant difference between the two years for specific leaf weight at the developmental stage (P<0.05), indicating that the specific leaf weight in soybean was easily affected by environmental conditions. Starter nitrogen fertilizer statistically increased specific leaf weight at early stage (V4, and R2), but had no significant impact on specific leaf weight at R4 stage in 2013. However, significant response of specific leaf weight to nitrogen, applied and incorporated at planting was observed at V4, R2, and R4 stages in 2014 (P<0.05). Enhancing specific leaf weight may improve leaf photosynthesis in soybean [49]. Thompson et al. [50] observed that increasing appropriate photosynthetic rate per leaf area may improve grain yield in soybean. Selection for high specific leaf weight in soybean may increase photosynthetic rate [50], which in turn may improve grain yield. To maximize total photosynthetic performance, and perhaps the grain yield, of a soybean cultivar, it would be necessary to maintain high specific leaf weight. Results obtain from our study showed that starter nitrogen fertilizer promoted specific leaf weight, and subsequently had positive effect on grain yield.

**Table 10:** Effects of nitrogen treatments on specific leaf weight at different stages (g m^-2^).

Nitrogen treatments	V4			R2			R4		
	2013	2014	Mean	2013	2014	Mean	2013	2014	Mean
N_0_	48.03b	38.06c	43.05b	49.56b	45.06b	47.31c	53.68	50.67c	52.18b
N_25_	50.31a	39.54bc	44.93ab	50.38b	47.13ab	48.76b	54.84	52.08bc	53.46a
N_50_	49.59a	45.78a	47.69a	52.12a	48.81a	50.47a	55.19	54.10a	54.65a
N_75_	44.27c	43.21ab	43.74b	49.28b	47.09ab	48.19bc	54.04	53.02ab	53.53ab
ANOVA Effect									
Nitrogen (N)	*	**	*	*	*	**	ns	**	*
Year (Y)	***			***			***		
N×Y	**			Ns			ns		

Means with same letter are not significantly different (P<0.05) using LSD Test.

***, **, *, and ns denote significance at 0.001, 0.01, 0.05 and not significant (P>0.05), respectively.

### Response of leaf area index to starter nitrogen fertilizer

Results from this study showed that starter nitrogen fertilizer had much significant effect on leaf area index at V4, and R2 stages (Table 11). Leaf area index was significantly affected at R4 stage in 2014 and did not show significant response to starter nitrogen fertilizer at R4 stage in 2013. That may be due to difference growing environmental conditions. Plant growth like LAI was positively correlated with grain yield [51, 52]. Starter nitrogen fertilizer increased soybean leaf area index and therefore had subsequent effect on grain yield in 2014 (Tables 11 and 4). Our results corroborate the previous studies which reported that a small amount of starter N at planting could be beneficial to early growth [8, 42]. In our study, N_50_ produced highest leaf area index at the developmental stages. High level of nitrogen treatment (N_75_) decreased the leaf area index. That is because N_75_ inhibited the root activity and decreased the dry root weight (Tables 5 and 6) compared to N_50_, and thus reduced the leaf area index.

**Table 11:** Effects of starter nitrogen fertilizer on leaf area index at different stages.

Nitrogen treatments	V4			R2			R4		
	2013	2014	Mean	2013	2014	Mean	2013	2014	Mean
N_0_	1.19d	1.13c	1.21d	5.59c	5.10c	5.35c	5.04	4.87b	4.96b
N_25_	1.28c	1.31b	1.30c	5.86b	5.31bc	5.59b	5.03	4.98a	5.01ab
N_50_	1.53a	1.42a	1.48a	5.99a	5.89a	5.94a	5.16	5.04a	5.10a
N_75_	1.39b	1.38ab	1.39b	5.91ab	5.56ab	5.74b	5.10	5.01a	5.06a
ANOVA Effect									
Nitrogen (N)	***	**	***	***	**	***	ns	**	*
Year (Y)	ns			***			**		
N×Y	*			ns			ns		

Means with same letter are not significantly different (P<0.05) using LSD Test.

***, **, *, and ns denote significance at 0.001, 0.01, 0.05 and not significant (P>0.05), respectively.

### Correlation analysis among traits

The correlation among traits was shown in Table 12. There was positively significant relationship between root activity and grain yield at V4, R2, and R4 stages with a correlation coefficient of 0.4852*, 0.5862**, and 0.5953**, respectively, indicating that an increase in root activity can help to enhance the grain yield. This finding was in agreement with the work of Zhang et al. [46]. However, significantly positive relationship was not found between dry root weight and grain yield. Moreover, there was positive relationship between root activity and leaf nitrogen yield at V4, R2, and R4 stages, with a correlation coefficient of 0.7621**, 0.8792**, and 0.4134*, respectively. This revealed that increasing root activity benefited the uptake of nitrogen.

**Table 12:** Correlation coefficients among grain yield, root activity, dry root weight, leaf area index, specific leaf weight, photosynthetic rate, chlorophyll content and leaf N yield.

Developmental stage	Parameter	Dry root weight	Root activity	Photosynthetic rate	Leaf area index	Leaf N yield	Chlorophyll content	Specific leaf weight
V4	Grain yield	0.0898	0.4852*	0.4281*	0.3957	0.4317*	0.4261*	0.4028*
	Specific leaf weight	0.4051*	0.3774	0.7429**	0.1267	0.2359	0.6734**	
	Chlorophyll content	0.534**	0.5877**	0.8558**	0.4032	0.437*		
	Leaf N yield	0.5793**	0.7621**	0.3914	0.7763**			
	Leaf area index	0.6135**	0.833**	0.3649				
	Photosynthetic rate	0.606**	0.5742*					
	Root activity	0.7339**						
	Dry root weight	1						
R2	Grain yield	0.0731	0.5862**	0.7514**	0.8153**	0.2904	0.4153*	0.4986*
	Specific leaf weight	0.6166**	0.4542*	0.4278*	0.4206*	0.548*	0.5347**	
	Chlorophyll content	0.3818	0.4389*	0.4268*	0.526**	0.471*		
	Leaf N yield	0.652**	0.8792**	0.5576**	0.594*			
	Leaf area index	0.2739	0.7513**	0.4164*				
	Photosynthetic rate	0.5058*	0.5228**					
	Root activity	0.5368**						
	Dry root weight	1						
R4	Grain yield	0.2134	0.5953**	0.826**	0.6515**	0.2325	0.4447*	0.4965**
	Specific leaf weight	0.241	0.576**	0.7767**	0.4846*	0.2909	0.0504	
	Chlorophyll content	0.0506	0.2475	0.2669	0.4624*	0.0215		
	Leaf N yield	0.1486	0.4134*	0.2908	0.1787			
	Leaf area index	0.1656	0.3159	0.5109*				
	Photosynthetic rate	0.0124	0.4424*					
	Root activity	0.1871						
	Dry root weight	1						

Data in the table are r-values

* and ** represent correlation at 0.05 and 0.01, respectively.

Positive correlation between chlorophyll content and photosynthetic rate was observed in our study. Our findings supported some researchers who reported positive relationship between chlorophyll content and photosynthetic rate [46, 53]. As shown in Table 4, nitrogen fertilizer applied at planting enhanced soybean grain yield. It may be attributed to the increase in the chlorophyll content and photosynthetic rate of soybean leaf. Positive relationship between chlorophyll content and grain yield was observed at V4, R2, and R4 stages with a correlation coefficient of 0.4261*, 0.4153*, and 0.4447*, respectively. A significant and positive relationship between chlorophyll content and grain yield was recorded by Zhang et al. [54]. The relationship between leaf N yield and grain yield was positive, but not significant. Therefore, we assume that the SPAD value (chlorophyll content) could provide a better and faster estimate of potential grain yield than leaf N yield at the early growth stage.

Our results showed a significant and positive relationship between photosynthetic rate and soybean grain yield at V4, R2, and R4 stages, with a correlation coefficient of 0.4281*, 0.7514**, and 0.826**, respectively. Nitrogen fertilizer applied at the time of seeding caused high photosynthetic rate, therefore increasing grain yield. An early published report of Brun et al. [55] confirmed the significant positive correlation with soybean grain yield. Dornhoff et al. [56] and Peet et al. [57] also found photosynthetic rate to be positively correlated with grain yield in soybean. Statistically significant relationship between root activity and photosynthetic rate was observed at the development stage in our study, indicating that increase of root activity can help to enhance the photosynthetic rate and thus grain yield.

There was significant and positive correlation between specific leaf weight and photosynthetic rate at V4, R2, and R4 stages, with a correlation coefficient of 0.7429**, 0.4278*, and 0.7767**, respectively in our study. Our results corroborated the research conducted by Bhagsari et al. [58] and Hesketh et al. [59], who reported a positive relationship between specific leaf weight and photosynthetic rate in soybean. Enhancing specific leaf weight may improve leaf photosynthesis and increase grain yield of soybean [49, 50]. Because there was significantly positive correlation between specific leaf weight and grain yield at V4, R2, and R4 stages (Table 12).

Our results demonstrated that there was positive correlation between leaf area index and grain yield at V4, R2, and R4 stages with a correlation coefficient of 0.3957, 0.8153*, and 0.6515**, respectively (Table 12). Leaf area index is an important parameter that influences grain yield of soybean [60–62]. Similarly, Sun et al. [52] reported that there was a significantly positive relationship between leaf area index and soybean grain yield at pod-filling stage and seed-filling stage. Sincik et al. [63] reported a significant relationship between grain yield and leaf area index.

## Conclusions

Environmental conditions during the growing season significantly affected soybean grain yield (P<0.05), with 2013 having higher grain yield. Starter nitrogen fertilizer significantly promoted grain yield in 2014. This significant response of grain yield to N supply in 2014 could be ascribed to a significant increase in root activity, photosynthetic rate, leaf area index, and specific leaf weight at the developmental stage (V4, R2, and R4) in our study. Applied nitrogen fertilizer at sowing significantly affected root activity, photosynthetic parameters at the early stage (V4, and R2), but significant response was not observed at R4 stage in 2013. That may be due to different precipitation at the late growing stage between two years. The grain yield increment due to N_25_, N_50_, and N_75_ was 40.39 kg ha^-1^, 77.93 kg ha^-1^, and 49.87 kg ha^-1^, respectively in 2013, and 18.71 kg ha^-1^, 83.27 kg ha^-1^, and 61.96 kg ha^-1^, respectively in 2014 compared to no N treatment. The difference in grain yield obtained in our study could be adequate to offset additional fertilizer cost. Therefore, it is noted that applying starter nitrogen fertilizer at the time of planting is within the current production practices. Our results proved that, it is necessary to apply a certain amount of starter nitrogen fertilizer when planting in China.
